# Scalp injury management by a maxillofacial surgeon in a low-resource hospital

**DOI:** 10.1186/s40902-020-00283-2

**Published:** 2020-12-09

**Authors:** Paul Frimpong, Truc Thi Hoang Nguyen, Edinam Salia Nimatu, Emmanuel Kofi Amponsah, Soung Min Kim

**Affiliations:** 1 Oral and Maxillofacial Microvascular Reconstruction LAB, Brong Ahafo Regional Hospital, P.O. Box 27, Sunyani, Ghana; 2 Department of Oral and Maxillofacial Surgery, Dental Research Institute, School of Dentistry, Seoul National University, 101 Daehak-ro, Jongno-gu, Seoul, 03080 South Korea

**Keywords:** Head and scalp injury, Accident and emergency unit, Low-resource hospital, Scalp layer, Scalp advancement flap

## Abstract

**Background:**

Head or scalp injury is a life-threatening and typically accidental human injury. Most medical departments require immediate medical treatment and proper treatment with specialized medical personnel and facilities. However, in low-resource environments, such as the rural region of West Africa, the authors have treated emergency trauma patients and provided immediate treatment despite lack of resources.

**Case presentation:**

We reviewed three cases of scalp injury patients, with representative clinical information, and used these cases to outline feedback on scalp trauma treatment based on the specialty knowledge of general and emergency surgeon.

**Conclusions:**

Oral and maxillofacial surgeons are medical specialists that can immediately diagnose and treat these scalp injuries based on their medical knowledge and experience with the maxillofacial region.

## Background

The scalp is the soft tissue envelope of the cranial vault and consists of five layers of skin, dense connective tissue, epicranial aponeurosis, loose areolar connective tissue, and periosteum [1, 2]. These scalp layers can be affected by accidents, benign or malignant tumors, and other necrosis-induced chemical agents or radiation. Thus, scalp injury could be classified as partial-thickness with intact periosteum or full-thickness with exposed cranium, exposed dura, or exposed cerebral tissue, according to injury depth and involved layers [1–3]. These defects could be classified according to anatomical site and size, as small when the size is less than 2 cm, medium when the size is 2 to 2.5 cm, and large when the size is more than 2.5 cm [3, 4].

The skin of the scalp has unique characteristics, with limited skin mobility, inelastic galea aponeurotic tissues, and dense hair follicles [4]; thus, it is difficult to close even a small defect. Most cases of medium or large defects could be covered using a unilateral or bilateral advancement flap after wide subperiosteal dissection. We present three representative scalp injury cases with scalp advancement flap reconstruction and our management of these cases in a low-resource health facility.

## Case series

From August 2014 to December 2018, more than 89 patients who were diagnosed with scalp injury visited two surgeons in the Department of Oral and Maxillofacial Surgery at Brong Ahafo Regional Hospital. We herein report three patients with frontoparietal scalp defect from motor vehicular accident and include clinical photographs with each patient’s description and treatment procedures. The patients or their parents provided written informed consent for surgical reconstruction and approved the use of their photographs in scientific publication. This case series report was evaluated and approved by the Institutional Review Board of Seoul National University (S-D20200021).

### Case 1: a 5-year-old child hit by a motorbike

A 5-year-old toddler who had received all his childhood immunization vaccines presented with scalp injury from impact with a speeding motorbike 48 h. Upon arrival at Essam Government Hospital, he was managed with antibiotics and pressure dressing to stop the bleeding from the scalp and later referred to Brong Ahafo Regional Hospital for expert management. The child did not lose consciousness, there was no bleeding from his ears or nose, and he had not previously undergone any surgery.

**Fig. 1:**
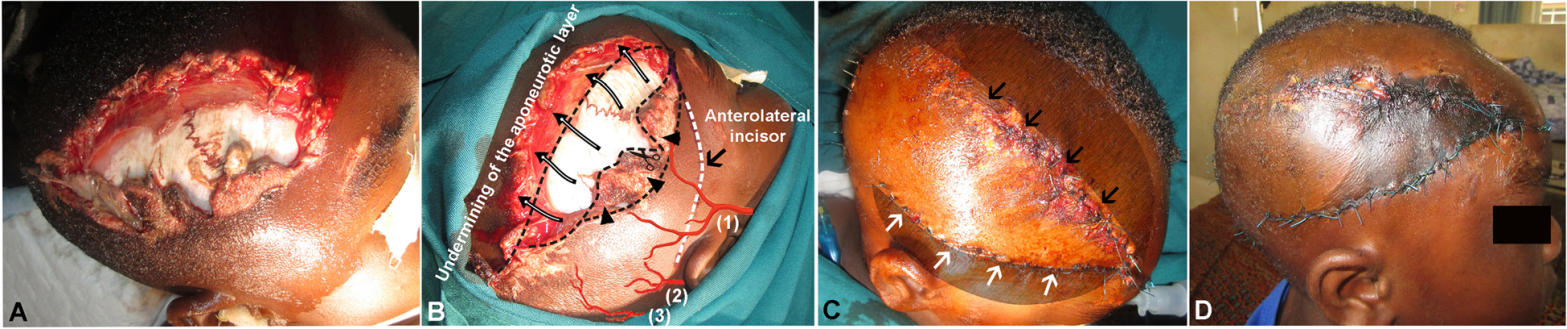
Clinical photos of case 1, a 5-year-old child that was hit by a motorbike. Pre-operative upper (**a**) and lateral (**b**) views showing exposed skull periosteum. The design of the advancement skin flap was showed; debridement of the infected tissue (black arrowheads), the anterolateral flap incision (white dotted line), the undermining of aponeurotic layer (long arrows), and scalp arteries including superficial temporal artery (1), posterior auricular artery (2), and occipital artery (3). Post-operative 1-day view with advancement skin flap coverage (**c**) showing appropriate closure suture of the primary wound (black arrows), closure suture of anterolateral flap incision (white arrows), and shadow marking of the undermining areas. **d** A view at 7 days’ post-operative

On presentation, the patient was a slightly febrile, moderately pale toddler that was well-hydrated, conscious, and alert, with a 3 × 8-cm-sized complete avulsion injury of the right scalp with scalloped margins extending from the right frontal region to the right parietal region exposing the underlying scalp (Fig. 1a). There was also an oblique 4-cm-sized laceration at the occipital scalp region; therefore, a clinical diagnosis of avulsion scalp injury with occipital laceration was made in the accident and emergency unit. The child was evaluated with complete blood count laboratory test, moved directly to the operation theater, and prepared for wound debridement with a primary wound closure operation.

Under general anesthesia with orotracheal intubation, the patient was placed in the supine position on the operation bed. Margins of avulsion and lacerations were shaved to about 3 cm from the edges of the wound, the scalp was washed thoroughly with about 100 ml of Savlon® (Johnson & Johnson, Bangladesh) antiseptic solution, and irrigation was performed with 1000 ml of normal physiologic saline (Fig. 1b). After excision of granulation and necrotic tissues, surgical markings were drawn, and incision sites were injected with 2 % lidocaine with 1:100,000 epinephrine (Huons, Seoul, Korea). A bilateral anteroposterior regional scalp flap was raised and advanced to cover the defect by careful dissection of underlying aponeurotic tissue to reduce raised flap tension. Then, non-absorbable 2-0 Nylon® (Ailee, Busan, Korea) suture material was used to oppose the subcutaneous edges of the wound to close the defect (Fig. 1c). Simple interrupted skin suturing was executed on the approximate flap without introducing tension within the flap. The wound was dressed with povidone-iodine solution and covered with sterile gauze and bandage. Antibiotic therapy was administered, and the wound was dressed daily. After continuous daily wound dressing for about 12 days, stitches were carefully removed; the patient had uneventful healing with a patch of alopecia occurring at the junction of the frontoparietal junction of the flap (Fig. 1d).

### Case 2: a 40-year-old female with a degloving scalp injury due to motorcycle accident

**Fig. 2:**
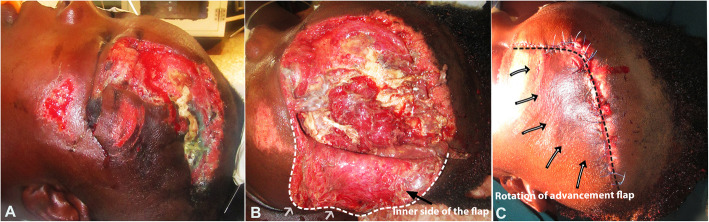
Clinical photos of case 2, a 40-year-old female with a degloving scalp injury due to a motorcycle accident. Pre-operative lateral view after washing (**a**). Exposed scalp structure at the skin degloving state showing the flap margin (white dotted line) with a minimized additional incision line (**b**), and the rotation and closure of the advancement skin flap (black dotted line and arrows) after wound debridement (**c**)

A 48-year-old female presented at the accident and emergency unit with an avulsion scalp injury after she was involved in a motor accident as an unrestrained passenger (Fig. 2a). She had a 10 × 12-cm-sized frontoparietal defect that was thoroughly debrided and irrigated with normal saline in the emergency unit.

The patient was moved immediately to the operation theater for delicate debridement with a primary closure operation under general anesthesia. The actual defect was revealed after thorough debridement to remove all necrotic tissue (Fig. 2b), and the surgical site was shaved with a number 11 surgical blade and marked. An inferior-based rotational advancement scalp flap was planned and designed, and the flap was incised and elevated in the loose connective tissue plan deep to the galeal aponeurosis. Careful incision of the galea was performed at regular minimal intervals to avoid iatrogenic injury to the scalp arterial blood supply. Approximation of the galea was conducted with monofilament absorbable 3-0 Vicryl® (Johnson & Johnson, Busan, Korea) suturing to ensure minimal tension on the wound. The flap was planned to be slightly longer than the defect, and bilateral advancement flaps were used to cover the main scalp defect (Fig. 2c).

**Fig. 3:**
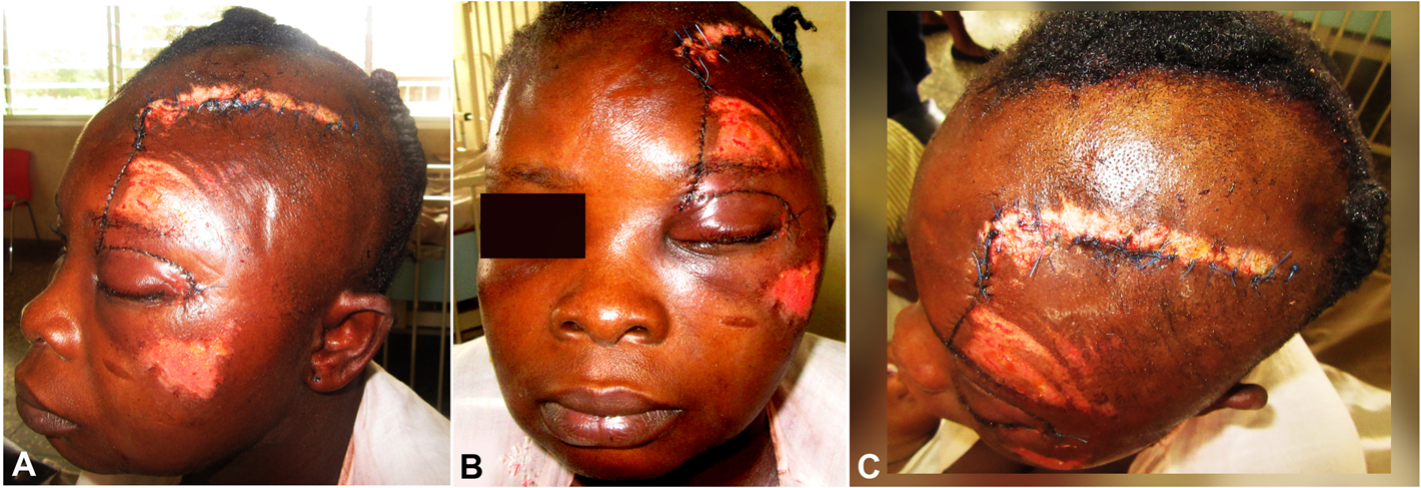
Clinical photos of case 2 at 7 days post-operative: lateral (**a**), frontal (**b**), and upper (**c**) views

**Fig. 4:**
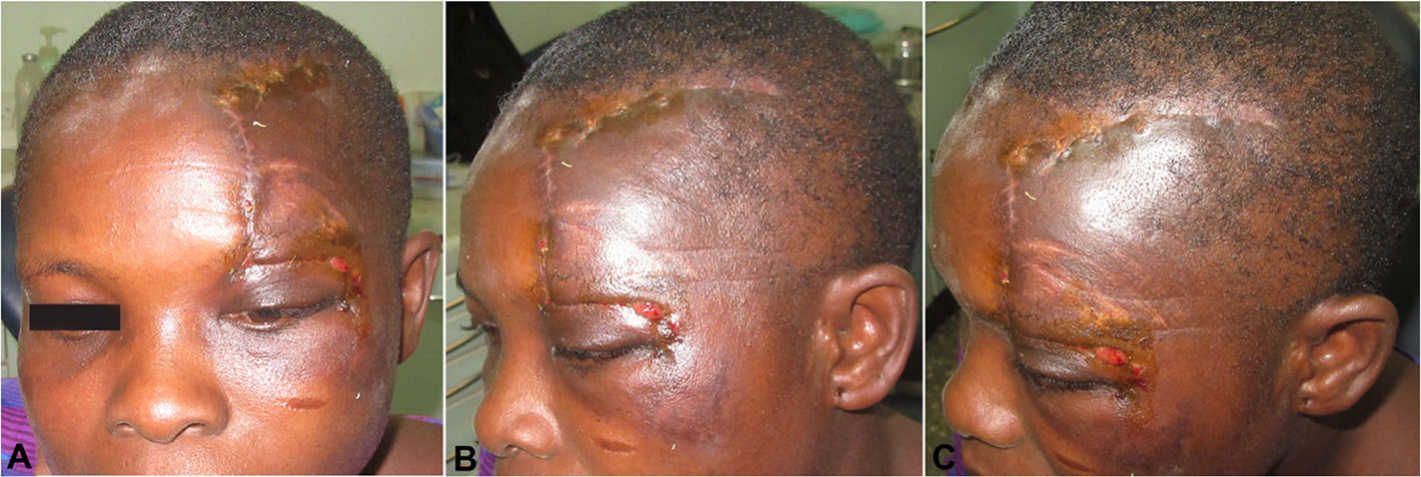
Clinical photos of case 2 at 40 days post-operative: frontal (**a**), lateral (**b**), and oblique upper (**c**) views

A pressure dressing was applied to the surgical wound immediately after surgery and removed after 1 day. Healing involved partial flap necrosis at the ipsilateral supraorbital region that was debrided and redressed daily. The patient was discharged from the hospital on the 7th postoperative day after daily wound management care (Fig. 3). After continuous wound dressing for about 14 days, stitches were carefully removed. The patient had uneventful healing without alopecia or loss of ear or eye function (Fig. 4).

### Case 3: a 55-year-old female with a depressed fronto-temporal bony fracture after being thrown off a motorbike

**Fig. 5:**
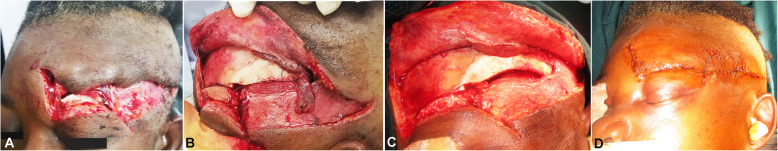
Clinical photos of case 3, a 55-year-old female with a depressed fronto-temporal bony fracture after being thrown off a motorbike. Pre-operative view showing frontal and temporal bone fractures with torn periosteum and subgaleal layers (**a**), cleaned state after debridement during surgery (**b**), and flap closure state after plate fixation and layer approximation (**c**, **d**)

A 55-year-old woman was rushed to the accident and emergency unit of Brong Ahafo Regional Hospital after being thrown off a motorbike on which she was riding as a passenger without a helmet. She lost but later regained consciousness upon arrival at the emergency unit. Detailed clinical examination revealed a middle-aged woman lying supine in bed, anicteric, afebrile, and not pale. She was not in respiratory distress, demonstrating adequate air entry into both lungs with a respiratory rate of 23 cpm, a blood pressure of 135/64 mmHg, a pulse rate of 82 beats/min, and a Glasgow Coma Scale of 15/15. She also had multiple facial abrasions with a degloving left scalp injury that extended from the frontal through the supraorbital to the left temporal region and an open depressed fronto-temporal skull fracture (Fig. 5a). A computed tomography scan of the head indicated an open fronto-temporal skull fracture and deep scalp laceration with bilateral maxillary sinus hemorrhage.

Thorough debridement of the degloving scalp injury was performed in the operation theater (Fig. 5b). Open reduction and direct fixation were performed using micro-miniplates and screws for the open depressed fronto-temporal skull fracture. Two-layered scalp closure was performed using a 2-0 multifilament polyglactic acid (Johnson & Johnson, Busan, Korea) and 2-0 Nylon® (Ailee, Busan, Korea) suture for connective tissue rearrangement and skin adaptation, respectively (Fig. 5c, d). A pressure dressing with head bandages was applied to the surgical wound. Stitches were removed after 12 days of continuous daily wound dressing; healing was uneventful, and she did not experience neurologic complications during the first 18 months of follow-up.

## Discussion

Scalp layers are comprised of the following components: (1) the skin, which is thick and hair-bearing and contains numerous sebaceous glands; (2) connective tissue, which is referred to as the superficial fascia, a fibro-fatty layer that connects the skin to the underlying aponeurosis and provides a passageway for nerves and blood vessels; (3) epicranial aponeurosis, or galea aponeurotica, which is a thin tendinous structure that provides an insertion site for the occipitofrontalis muscle; (4) loose areolar tissue, which loosely connects the epicranial aponeurosis to the pericranium and allows the superficial three layers of the scalp to move over the pericranium; and finally, (5) pericranium, which is the periosteum of the skull bones and is continuous with the endosteum [1–4].

Reconstruction of a scalp injury is difficult due to the complexity of the underlying skeleton, inelasticity of the scalp skin such as the galea aponeurotica and paucity of adjacent tissues [3]. Basically, would closing starts from approximation of the aponeurotic layer with continuous absorbable suture materials, and then full-thickness simple or mattress sutures could be considered and combined with hemostasis. However, if there is a scalp defect from severe trauma or an accident, representative rotation or advancement along the loose areolar tissue is recommended and occurs in most cases [5–7]. Our presented cases had large-sized defect in the anterior and lateral scalp region, for which a sliding advancement flap after wide undermining was recommended. Although a full-thickness skin graft or other skin grafts, including hair follicles, are treatment options, these procedures were not possible in the low-resource hospital environment in this article.

The rural areas of Ghana, West Africa, lack medical facilities or medical support items and do not employ maxillofacial specialties. Although accurate statistics are not available, many disadvantages, including traffic and climatic conditions including repeated heavy rain during the rainy season, can result in fatal accidents and increase the number of life-threatening facial injuries. In addition, terminal scalp hair characteristics differ according to ethnicity, such as diameter, cross-sectional shape, and general appearance [8]. The hair of those with African ethnicity typically has an elliptical and ribbon-like shape with a curly appearance, which is advantageous during scalp injury management compared with straight or wavy characteristics typical of Asians or Caucasians [https://en.wikipedia.org/wiki/Hair_follicle].

Regardless of scalp skin thickness, excessive tension should be considered [7], and a rotational flap with or without advancement based on vascular anatomy could be considered [9]. Bilateral advancement flaps for reconstructing medial or large scalp defects could be considered, as in our cases. Additionally, accurate debridement of devitalized tissue with correct approximation of epicranial aponeurosis should be confirmed in every scalp injury patient. In literature, the recommended timing of stitch removal in the scalp is 7 to 10 days [10]. In our cases, the stitches were removed after 10 to 14 days to ensure the adequate healing of the wound. The follow-up visit was made at 1 week; 1, 3, 6 months; and 1 year and 2 years postoperative.

Due to the circumstances, randomized controlled trials cannot be conducted in these emergency situations, and sterile irrigation is not sufficient for cleaning wounds of debris but does dilute bacterial load before closure in scalp injury management. A Cochrane review supports the use of potable tap water, as opposed to sterile saline, for wound irrigation [11]. In addition, the use of clean nonsterile gloves, rather than sterile gloves, during wound repair management has little influence on the rate of subsequent wound infection. At presentation, lacerations are considered to be contaminated, and physicians should make every effort to avoid introducing additional bacteria to the wound [11]. In most cases, physicians wear protective masks and gloves to try and maintain strict sterile environments; therefore, to further understand patient history, physicians and hospital staff should try to obtain a detailed history, including tetanus vaccination and previous allergies, and determine the timing and situational circumstances to optimize treatment of scalp injury patients.

## Conclusions

Scalp injury management is considered very difficult, and the procedures demand careful attention to perform quick and accurate diagnoses and operations. Patients with wide, large scalp defects located in the frontal or parietal regions cannot achieve primary closure without a wide undermining. Oral and maxillofacial surgeons should be active and prepared to assist in any situation as specialists who can immediately diagnose and treat these head injuries based on their extensive knowledge and experience with the maxillofacial skin, muscles, and masticatory muscles. Fundamental management of these injuries is very important to optimize patient care and outcomes.

## Data Availability

Data sharing is not applicable to this article as no data sets were generated or analyzed during the current study.
